# Epidemiology of bronchiolitis: a description of emergency department visits and hospitalizations in Puerto Rico, 2010–2014

**DOI:** 10.1186/s41182-017-0064-7

**Published:** 2017-10-02

**Authors:** Andrea Rivera-Sepulveda, Enid J. Garcia-Rivera

**Affiliations:** 1 Division of Pediatric Emergency Medicine, Department of Pediatrics, Saint Louis University School of Medicine, 1402 S. Grand Boulevard – Glennon Hall, Room 2717, 63104 Saint Louis, MO USA; 2 School of Health Professions, University of Puerto Rico Medical Sciences Campus, and School of Medicine, San Juan, Puerto Rico; 3 Endowed Health Services, University of Puerto Rico School of Medicine, Medical Sciences Campus, San Juan, Puerto Rico

**Keywords:** Bronchiolitis, Pediatric, Children, Epidemiology, Hospitalization, Puerto Rico, Respiratory syncytial virus, Trend

## Abstract

**Background:**

Little is known about the epidemiology of bronchiolitis as a clinical diagnosis and its impact on emergency department visits and hospitalizations in tropical and semitropical regions. We described the epidemiology of bronchiolitis emergency visits and hospitalizations, its temporal trend and geographic distribution in Puerto Rico between 2010 and 2014.

**Methods:**

We performed a retrospective descriptive analysis of a representative sample of privately insured children with bronchiolitis from January 2010 to December 2014. Data was provided by the largest private health insurer in Puerto Rico and identified children < 24 months of age with bronchiolitis by *International Classification of Diseases, Ninth Revision* code 466, 466.11, and 466.19. Chi-square and one-way ANOVA compared sex, age, diagnosis, and severity across the years. Joinpoint Poisson regression analysis evaluated the temporal trend distribution of bronchiolitis hospitalizations per calendar year. A *P* value less than 0.05 was statistically significant.

**Results:**

During the study period, the annual proportion of emergency department visits and hospitalizations due to bronchiolitis increased from 3 to 5%, and 26 to 38%, respectively. The annual incidence rate of hospitalizations was 3.2 per 1000 privately insured children < 24 months. Non-RSV bronchiolitis was the most frequent diagnosis (51%). Hospitalizations occurred year-round, but increased significantly from August through December. Most children hospitalized resided in the metropolitan San Juan (35%) and surrounding urban areas. Total hospital charges decreased from $3.78 to $3.74 million, with an average cost per hospitalization of $4320.12 (11.3% increase; *P* = 0.0015).

**Conclusions:**

This is the first study that evaluates the epidemiological characteristics of bronchiolitis in a primarily Hispanic population, living in a tropical country, and using data from a privately insured population. We found a small but significant increase in proportion of emergency visits and hospitalizations. Temporal trend shows year-round hospitalizations with an earlier seasonal peak and longer duration, consistent with Puerto Rico’s seasonal rainfall throughout the study period. Further studies are needed to elucidate whether this epidemiologic pattern can also be seen in publicly insured children and whether Hispanic ethnicity is a risk factor for increased hospitalizations or is related to health disparities in the US healthcare system.

## Background

Bronchiolitis constitutes an important public health burden in the pediatric population worldwide [1–4], as the leading cause of emergency department visits and hospitalizations in infants younger than 2 years of age [5]. In the USA, emergency department visits and hospitalizations due to bronchiolitis have increased steadily over the last 30 years [6, 7], incurring in over 150,000 emergency visits and hospitalizations annually, and exceeding $1.7 billion in combined charges [1, 5, 7, 8]. Clinical manifestations associated with disease severity are influenced by sex, age, previous bronchiolitis, co-morbidities, environmental exposure, and the host’s immune response to the infection [9]. About 1 in 9 infants develop bronchiolitis in the first year of life, classifying it as a major cause of clinical morbidity and rising inpatient healthcare costs [10, 11]. This is influenced by the significant variation in the frequency and type of intervention [12, 13], as well as healthcare resource utilization [14] and disparities in the healthcare system [15].

Past studies on bronchiolitis hospitalizations have focused on those caused by the respiratory syncytial virus (RSV), the most common etiologic agent [16]. In temperate climates, late fall and winter epidemics of bronchiolitis are usually linked to RSV, with slight variability in seasonal onset and duration [2, 16–19]. In tropical and semitropical climates with warm temperatures and seasonal rainfall like Puerto Rico, RSV occurs throughout the year [20], usually with outbreaks during the rainy season [9, 19–21]. This suggests that RSV bronchiolitis has unique epidemiologic characteristics depending on geographic region and climate [9, 21, 22].

Little is known about the epidemiology of bronchiolitis as a clinical diagnosis, with disregard as to the etiologic agent, and its impact on emergency visits and hospitalizations in tropical and semitropical regions like Puerto Rico. Therefore, the objectives of this study are to (1) describe demographic characteristics of emergency department visits due to bronchiolitis, (2) describe demographic and clinical characteristics of patients hospitalized with bronchiolitis, (3) examine temporal trend distribution of bronchiolitis hospitalizations, and (4) describe geographic distribution of bronchiolitis hospitalizations in Puerto Rico.

## Methods

### Study design

This retrospective descriptive study analyzed secondary data provided by the largest private health insurer in Puerto Rico. During the study period, two thirds of the population was covered under public health insurance; approximately one third under private health insurance. The insurer under study served an estimated average of 680,139 participants per year from 2010 to 2014, over 50% of privately insured Puerto Ricans on a yearly basis.

### Data source

The insurer provided two aggregated and de-identified data sets: (1) children under 24 months of age who visited an emergency department in Puerto Rico from January 1, 2010, to December 31, 2014; and (2) children under 24 months of age with a primary or secondary diagnosis for acute bronchiolitis upon hospitalization in Puerto Rico from January 1, 2010, to December 31, 2014. Due to insurer data extraction procedures and codification, the databases for the emergency department visits and hospitalizations were mutually exclusive and independently analyzed.

### Identification of sample

The diagnosis of bronchiolitis was defined using the *International Classification of Diseases, Ninth Revision, Clinical Modification* (ICD-9-CM) codes for acute bronchitis and bronchiolitis (ICD-9-CM: 466), acute bronchiolitis due to RSV (ICD-9-CM: 466.11), and acute bronchiolitis due to other infectious organism (ICD-9-CM: 466.19). An emergency visit due to bronchiolitis was defined as any patient < 24 months evaluated at the emergency department with a discharge diagnosis of bronchiolitis based on the ICD-9-CM code. A hospitalization due to bronchiolitis was defined as any patient < 24 months admitted with a primary or secondary diagnosis of bronchiolitis upon hospitalization based on the ICD-9-CM code. Severe bronchiolitis was defined as a hospitalization that required admission to the Pediatric Intensive Care unit (PICU).

### Study variables

Emergency department data included (1) total visits per year by age group (months) and sex, and (2) total visits due to bronchiolitis per year by age group (months) and sex. Hospitalization data included (1) total number of patients hospitalized per year by age group (months) and sex, (2) number of hospitalizations due to bronchiolitis per year, (3) number of cases by diagnosis (ICD-9-CM code), (4) number of hospitalizations per month of admission, (5) number of hospitalizations per unit (general versus intensive care), (6) mean length of stay per month, (7) hospital charges per day based on number of patients hospitalized per year, and (8) number of hospitalizations per health insurance region based on municipality of residence.

Daily precipitation (inches) was monitored and provided by the National Oceanic and Atmospheric Administration from the primary weather station at the San Juan International Airport [23]. Total hospital charges reflected the total facility fees reported for the aggregated hospitalizations per year. Cost information was obtained from the insurer’s billing reports. The geographical distribution of bronchiolitis hospitalizations were aggregated into seven health regions based on the categories used by the insurer and the Puerto Rico Department of Health.

### Outcome measures

The primary outcome measures were (1) annual proportion of emergency department visits due to bronchiolitis, (2) annual proportion and annual incidence rate of bronchiolitis hospitalizations, and (3) temporal trend distribution of bronchiolitis hospitalizations per calendar year. Other outcomes of interest included disease severity, hospital charges, and proportion of bronchiolitis hospitalizations by geographic region.

### Statistical analysis

Data was analyzed using descriptive statistics, including frequency distribution and measures of association. Categorical variables were analyzed using frequency and percentages; continuous variables were analyzed using means and standard deviation. To estimate the burden of bronchiolitis in this population, we calculated the annual proportion of bronchiolitis emergency visits among total emergency visits per year by age group, and the annual proportion of bronchiolitis hospitalizations among total hospitalizations per year by age group. The denominator of sex and age group distribution for bronchiolitis hospitalizations differed from ICD-9-CM codes and hospital unit due to post-adjustment measures provided by the insurer for billing purposes that included multiple admissions incurred by a single patient, coding, billing, and claim processing. To estimate the annual incidence rate of bronchiolitis hospitalizations per 1000 insured participants by age group, we used the insurer’s enrollment data from June 2014; taking into consideration an adjustment for the annual fluctuation in health insurance enrollment with a stable descent of 13% since 2012 to estimate the insured population per year. To estimate the burden of economic inflation and facilitate direct comparisons between years for hospital charges in Puerto Rico and the USA, we used the medical care component of the Consumer Price Index [24]. Chi-square was used to evaluate if there were differences between sex, age, diagnosis, and severity distribution over time. One-way ANOVA was used to evaluate if there were differences between hospital charges and length of stay across the years. Temporal trend distribution of bronchiolitis hospitalizations per calendar year was evaluated by Joinpoint Regression Program, Version 4.2.0.2 June 2015 (Statistical Methodology and Applications Branch, Surveillance Research Program, National Cancer Institute) [25]. Joinpoint Poisson regression analysis consists of a series of permutation tests that define points in time when the trend changed significantly. The estimated annual percentage change is represented by the magnitude of change in slopes within the accumulated monthly proportion of bronchiolitis hospitalizations per year in the time trend, alongside its 95% confidence intervals. We evaluated the total bronchiolitis hospitalizations by the month of diagnosis for the period of 5 years. A descriptive analysis was performed to evaluate the association between total precipitation (inches) and total bronchiolitis hospitalizations per month and year; and the geographic distribution of bronchiolitis hospitalizations per health region. A *P* value less than 0.05 was established as statistically significant. Analyses used STATA version 11 (Stata Corporation, College Station, TX, USA).

This study was approved by the University of Puerto Rico, Medical Sciences Campus (IORG000223) Institutional Review Board.

## Results

### Emergency department visits due to bronchiolitis

A total of 48,886 emergency department visits of children < 2 years were identified from 2010 to 2014, of which 2281 (4.7%; SD 1%) were due to bronchiolitis (Table 1). During the study period, there was a significant difference in the annual proportion of bronchiolitis emergency visits (*P* < 0.001). Infants < 12 months of age represented 68% of all bronchiolitis emergency visits. Subgroup analyses showed that the 3.1- to 6-month age group was most frequently diagnosed with acute bronchiolitis (12%; SD 1.6%) and represented 21% (SD 2.5%) of all bronchiolitis emergency visits. During the study period, the annual proportion of bronchiolitis emergency visits among sexes (*P* = 0.15), and age groups (*P* = 0.116) were relatively constant.

**Table 1:** Distribution of emergency department visits and hospitalizations due to bronchiolitis, 2010–2014

	Years, n (%)					
	2010	2011	2012	2013	2014	*P* value
Emergency department visits						
Total	12,515	10,609	10,403	8549	6810	
Due to bronchiolitis	434 (3)	553 (5)	525 (5)	423 (5)	346 (5)	< 0.001
Sex^a^						
Male	223 (3)	316 (6)	296 (5)	247 (5)	206 (6)	0.15
Female	211 (4)	237 (5)	229 (5)	176 (5)	140 (5)	
Age distribution^b^						
0 to 3 months	44 (6)	35 (7)	51 (11)	28 (8)	28 (9)	0.116
3.1 to 6 months	96 (10)	137 (14)	101 (12)	84 (13)	67 (11)	
6.1 to 9 months	94 (6)	119 (9)	121 (9)	89 (9)	74 (9)	
9.1 to 12 months	83 (5)	89 (6)	86 (6)	76 (6)	50 (6)	
12.1 to 24 months	117 (2)	173 (3)	166 (3)	146 (3)	127 (3)	
Hospitalizations						
Total	3624	3052	2604	2467	2086	
Due to bronchiolitis	941 (26)	1209 (40)	1076 (41)	969 (39)	791 (38)	< 0.001

^a^Percentage is based on annual proportion of bronchiolitis emergency visits among total emergency visits per sex

^b^Percentage is based on annual proportion of bronchiolitis emergency visits among total emergency visits per age group in months

### Demographics and selected characteristics of hospitalized children with bronchiolitis

A total of 13,833 hospitalizations were reported from 2010 to 2014, of which 4986 were due to bronchiolitis; corresponding to 37% (SD 6%) of all hospitalizations for children < 24 months (Table 1). Although the total number of bronchiolitis hospitalizations decreased from 941 in 2010 to 791 in 2014, the annual proportion of bronchiolitis hospitalizations showed a small but significant increase during the study period (*P* < 0.001). Bronchiolitis hospitalizations were most frequent in males (59 versus 41%, *P* = 0.019) and children < 1 year of age (62%; SD 13%) (Table 2). Subgroup analyses showed that the 3.1- to 6-month age group had the highest percentage of bronchiolitis hospitalizations (20%; SD 9%) during the study period, after which the hospitalization percentage declined steadily by age group (*P* < 0.001). We estimated the annual incidence rate of bronchiolitis hospitalizations as 3.2 per 1000 privately insured children < 24 months of age.

**Table 2:** Demographics and characteristics of bronchiolitis hospitalizations, 2010–2014

	Years, n (%)					
Characteristics	2010	2011	2012	2013	2014	*P* value
Demographics	(*n* = 941)	(*n* = 1209)	(*n* = 1076)	(*n* = 969)	(*n* = 791)	
Sex^a^						
Male	565 (32)	705 (41)	586 (42)	582 (42)	485 (40)	0.019
Female	376 (20)	504 (37)	490 (41)	387 (36)	306 (35)	
Age distribution^b^						
0 to 3 months	124 (14)	176 (41)	134 (40)	88 (34)	91 (39)	< 0.001
3.1 to 6 months	190 (44)	289 (58)	233 (59)	120 (39)	169 (55)	
6.1 to 9 months	183 (43)	225 (54)	213 (56)	93 (30)	125 (45)	
9.1 to 12 months	137 (34)	153 (40)	158 (46)	80 (29)	116 (42)	
12.1 to 24 months	307 (21)	366 (28)	338 (29)	588 (44)	290 (29)	
Cost per hospitalization						
Mean	$4016	$4313	$4269	$4277	$4725	0.0015
Standard deviation	$3215	$2812	$3606	$3794	$3128	
Cost per day (mean)	$852	$865	$851	$910	$948	
Total charges per year	$3,779,161	$5,214,823	$4,593,615	$4,144,186	$3,737,647	
Hospitalizations	(*n* = 924)	(*n* = 1219)	(*n* = 1140)	(*n* = 969)	(*n* = 798)	
ICD-9-CM Code						
466	61 (7)	52 (4)	55 (5)	64 (7)	38 (5)	< 0.001
466.11	254 (27)	603 (50)	601 (53)	391 (40)	363 (45)	< 0.001
466.19	609 (66)	564 (46)	484 (42)	514 (53)	397 (50)	
Hospital unit						
General Ward	900 (97)	1183 (97)	1105 (97)	949 (98)	772 (97)	0.54
PICU	24 (3)	36 (3)	35 (3)	20 (2)	26 (3)	
Length of stay						
Total days	4436	6028	5396	4553	3942	
Mean (SD)	4.8 (3.2)	4.9 (2.8)	4.7 (3.2)	4.7 (3.6)	4.9 (3.6)	0.42

*ICD-9-CM* international classification of disease, *9th revision* clinical modification, *466* acute bronchitis and bronchiolitis, *466.11* acute bronchiolitis due to Respiratory Syncytial Virus; *466.19* acute bronchiolitis due to other infectious organism, *PICU* Pediatric Intensive Care Unit, *SD* standard deviation

^a^Percentage is based on annual proportion of bronchiolitis hospitalizations among total hospitalizations per sex

^b^Percentage is based on annual proportion of bronchiolitis hospitalizations among total hospitalizations per age group in months

### Bronchiolitis-related diagnoses

There were 5050 hospitalizations with a diagnosis of acute bronchiolitis upon hospitalization (Table 2). We found a significant change in the distribution of hospitalizations for acute bronchitis and bronchiolitis (5.3%; SD 1%), acute bronchiolitis due to RSV (43.8%; SD 10%) and acute bronchiolitis due to other infectious organism (50.9%; SD 9%) during the study period. The proportion of hospitalizations attributed to RSV bronchiolitis increased from 27% in 2010 to 45% in 2014, while the proportion of hospitalizations due to non-RSV bronchiolitis remained the most frequent diagnosis during the study period.

### Temporal distribution of bronchiolitis hospitalizations

**Fig. 1:**
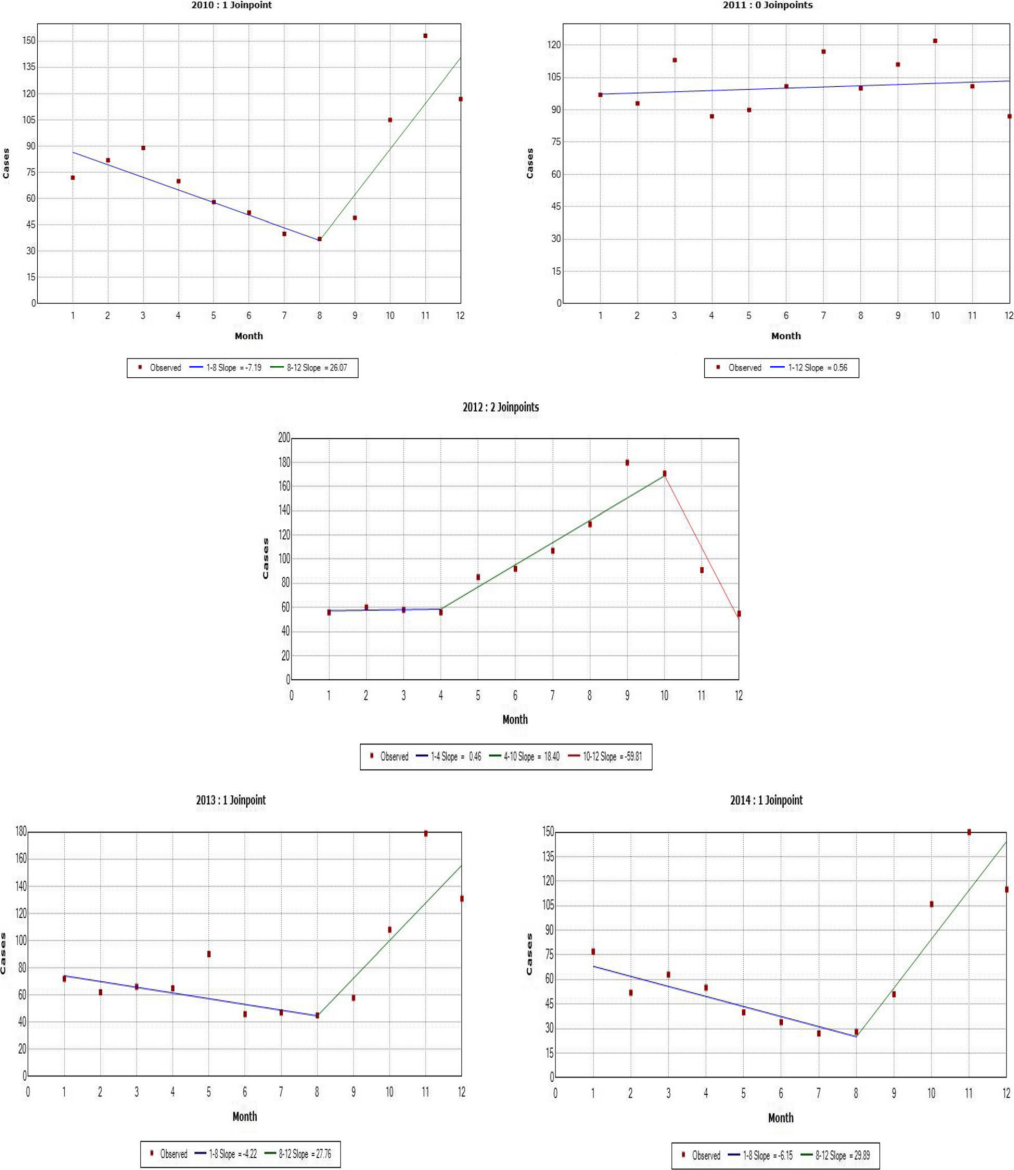
Joinpoint regression analysis of monthly cumulative frequency of bronchiolitis hospitalizations by year, 2010–2014

**Fig. 2:**
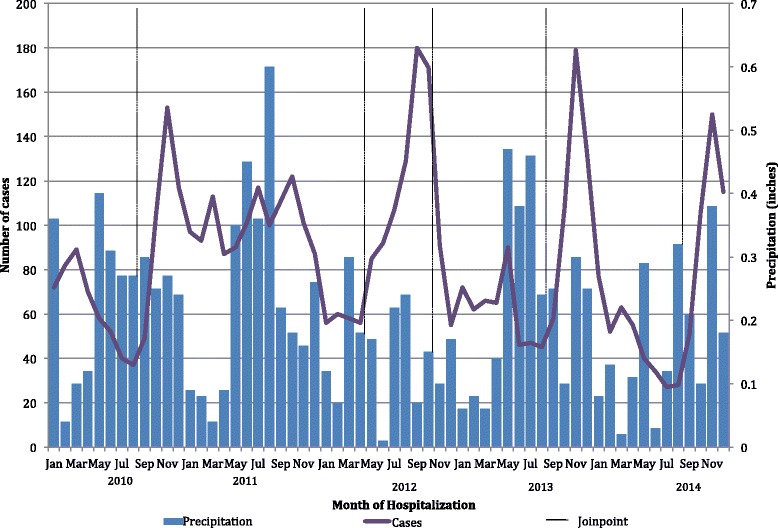
Association between monthly cumulative frequency of bronchiolitis hospitalizations and rainfall, 2010–2014

Joinpoint regression analysis showed a significant increase in bronchiolitis hospitalizations as of August, with an increasing trend until December in the years 2010, 2013, and 2014 (Fig. 1). The year 2011 did not show any discernible trend, while 2012 showed a significant early increase in trend from April to October. Bronchiolitis hospitalizations occurred throughout the year, increasing in September through December, with 44% (SD 7%) of bronchiolitis hospitalizations occurring during this 4-month period. The years 2011 and 2012 had an earlier increase in bronchiolitis hospitalizations above the yearly mean hospitalization percentage, starting in July and ending in October, for a cumulative hospitalization proportion of 37% (SD 1%) and 52% (SD 3%), respectively, (Fig. 2). Temporal analysis between bronchiolitis hospitalizations and amount of rainfall did not show any discernible distribution. However, a pattern of heavy cumulative rainfall representing over half (55%) the total amount of precipitation per year (1.4 inches; SD 0.54 inches) was seen during May through September, before each hospitalization surge, and 58% (SD 4.8%) of bronchiolitis hospitalizations occurred as of July through December, the start of the rainy season in Puerto Rico. Official data from a single weather station for the years 2010–2014 show a mean annual rainfall of 2.47 inches (SD 0.54 inches).

### Bronchiolitis severity

Of the 4986 children, 91% had a single hospitalization, 8% had two hospitalizations, and 1% had three or more (*P* < 0.001). The proportion of severe bronchiolitis hospitalizations (3% PICU hospitalization rate, *P* = 0.54) and the mean length of stay (4.8 days, *P* = 0.42) remained stable throughout the study period.

### Healthcare costs

Between 2010 and 2014, the mean cost per day increased 11.3% from $851.93 to $948.16, with an average cost of $4,320.12 per hospitalization, adjusted for inflation (1.7% mean yearly increase; *P* = 0.0015). Total hospital charges per year for bronchiolitis hospitalizations decreased from $3.78 to $3.74 million (Table 2).

### Geographical distribution of bronchiolitis hospitalizations

For the purpose of this analysis, Puerto Rico was divided into seven health regions based on municipality of residence. Hospitalizations were most frequent in the San Juan metropolitan area (35%; SD 1%), followed by the urban area of Bayamón (24%; SD 2%).

## Discussion

We identified emergency department visits and hospitalizations due to bronchiolitis within a representative sample of privately insured children < 2 years between 2010 and 2014. Our study shows an annual proportion of emergency visits due to bronchiolitis of 5% (SD 1%) and an annual proportion of bronchiolitis hospitalizations of 37% (SD 6%). We estimated an annual incidence rate of 3.2 bronchiolitis hospitalizations per 1000 privately insured children < 2 years of age within the general population. Bronchiolitis hospitalizations occurred year-round, increasing significantly as of August through December, and taking place more frequently in the main Metropolitan area of Puerto Rico.

During the study period, we identified more than 2200 emergency visits by children with bronchiolitis. Our data shows a similar annual proportion of bronchiolitis emergency visits to other studies in the USA. A study by Hasegawa et al. [26] based on a US nationally representative sample of 1,435,110 emergency visits with bronchiolitis had a similar (4.3%) annual proportion of bronchiolitis visits among total emergency visits. However, a study by Carroll et al. [27] based on a retrospective cohort of 103,670 infants enrolled in Tennessee Medicaid, reported that during the first year of life, 6.2% of infants had an emergency visit due to bronchiolitis, with African-American and Latino infants being more likely to have such visits than white infants. The higher proportion of bronchiolitis emergency visits found in the Tennessee study may be explained by the inclusion of children under public insurance coverage, which is a risk factor for increased bronchiolitis incidence and suggests a differential use of medical services [26, 27].

Previous studies have shown that in the USA, an increase in bronchiolitis hospitalizations was observed during the 1990s [6, 7] and early 2000s [2, 27], with a decrease in the mid-to-late 2000s in both the USA [1] and Spain [18]. Our study shows an annual proportion of bronchiolitis hospitalizations to be twice as high as that reported in a study by Hasegawa et al. (16%) [1]. Some studies, though not looking at incidence rate of hospitalizations due to bronchiolitis, have shown that Hispanic infants are twice as likely to be hospitalized for bronchiolitis than white infants [28, 29]. A study by Mansbach et al. [7], evaluating 1,868,000 emergency visits for children younger than 2 years of age, established that Hispanic ethnicity independently predicts both emergency visits and hospitalization. Another study of healthcare resource utilization among Latino infants in the USA showed that out of 674 previously healthy infants with acute respiratory illness, Latinos had lower thresholds for hospitalization than non-Hispanic whites and African Americans [14]. Authors suggested that this could be due to access to healthcare resources and language barriers. Our data shows that the annual proportion of bronchiolitis hospitalizations in Puerto Rico increased between 2010 and 2014, with a small but statistical significance. This however, does not equate to an increased incidence of bronchiolitis hospitalizations. This significant variation could be explained by a change seen from the year 2010 to 2011, when there was a 14% annual proportion increase in bronchiolitis hospitalizations. Variations such as the one seen in our findings have been associated with changes in disease incidence and severity, as reported in a previous study [1]. Our study showed that the proportion of severe bronchiolitis remained constant throughout the study period and was similar to other studies [30–36]. Also, the highest annual proportion of bronchiolitis hospitalizations was consistent with the US peak bronchiolitis hospitalization, to be between 2 and 6 months of age [5, 6, 17, 32, 36–39]. This evidence suggests that age < 6 months may be a non-medical risk factor for bronchiolitis hospitalization in this population [9, 40].

During the study period, the proportion of RSV bronchiolitis hospitalizations varied significantly from 27 to 45%, with a 23% annual proportion increase from the year 2010 to 2011. One Canadian study reported the emergence of a new RSV variant, namely ON1 genotype (subgroup A), in late 2010 [41], associated with an altered immunogenicity and pathogenicity of the virus. Furthermore, after gradually spreading worldwide, one study described that the emergence of ON1 genotype was associated with an increased lower respiratory tract infection incidence and severity [42]. Although we lack information on the RSV genotype within our study population, the emergence of this new RSV variant in the current study site may also be important in the increase of RSV-attributed bronchiolitis hospitalizations. Overall, non-RSV bronchiolitis was more frequently diagnosed than RSV bronchiolitis, with the exception of 2011 and 2012. This finding is consistent with the RSV surveillance data provided by the Puerto Rico Department of Health [43], who reported that in 2011 and 2012, positive RSV tests doubled. This is different from studies in temperate areas, where it has been reported that RSV accounts for 50–80% of yearly bronchiolitis cases [39, 44, 45]. This may also be due to variations in codification processes and availability of diagnostic analyses such as antigen testing, virus isolation, and/or polymerase chain reaction (PCR) tests at admitting hospitals. Because laboratory confirmation for RSV is widely available in Puerto Rico, the ICD-9-CM code for acute bronchiolitis due to RSV (466.11) is only attributed and billed when there is evidence of RSV testing in the medical record. We assumed that if a hospital was unable to carry out this testing, the record should have an ICD-9-CM code 466 or 466.1 for acute bronchiolitis; the latter of which was not included in this study, as the data was not provided by the insurer. Because the ICD-9-CM code 466 represents an average of 5% of cases per year, we performed a secondary analysis between the ICD-9-CM codes 466.11 and 466.19, excluding 466, which did not change the significance of the distribution of diagnoses from 2010 to 2014.

Joinpoint regression analysis from 2010 to 2014 showed bronchiolitis hospitalizations year-round, with an increase during September through December. During the years 2011 and 2012, there was an increase in bronchiolitis hospitalizations from July through October, consistent with the reported RSV epidemic [43]. This was also consistent with the findings of the National Surveillance System that included the RSVAlert® program and the National Respiratory and Enteric Virus Surveillance System who determined that in Puerto Rico, RSV activity is perennial and lacks a discernible seasonal pattern [46], with an increased activity in RSV usually seen from September through December [47]. This seasonal trend is most closely related to the US Department of Health and Human Services (HHS) Region of Florida, which presents an earlier RSV season onset and longer duration than the rest of the USA [16]. The pattern of bronchiolitis hospitalizations suggests an association with Puerto Rico’s seasonal rainfall (Fig. 2) [23], but we were not able to establish a statistical relationship. This may be because we are evaluating the relationship between a limited set of climate indicators and bronchiolitis as a clinical diagnosis and not as the result of an etiologic agent. Because a causative agent can be transmitted in different ways according to their atmospheric circumstance, thereby affecting the behavior of these infectious particles [48], it becomes important to elucidate the interaction between other environmental components and the transmission of the disease.

The decrease in total hospital charges during the study period is associated with a decrease in bronchiolitis hospitalizations and total hospital days. The cost of bronchiolitis hospitalizations were on average over 50% higher than the US $2815 geometric mean, reported by Hasegawa et al. [1] in 2009. The reason for this marked difference may be associated to hospital mean length of stay, which is twice as high in Puerto Rico.

In our study, most hospitalizations occurred in patients from the two main metropolitan areas, which together make up over half of bronchiolitis hospitalizations during the study period. This finding most likely reflects the socioeconomic status of privately insured population, with a higher number of subscribers living in the metropolitan area and surrounding urban and suburban municipalities, greater number of hospitals, urbanization, and industrialization [49]. One study however, reported that infants in rural and suburban areas were more likely to have a diagnosis of bronchiolitis and increased risk of higher severity when compared to infants in urban areas [27]. Because we lack information about the insurer’s subscribers per municipality, month or year, we were unable to calculate the annual proportion of bronchiolitis hospitalizations by geographic region.

### Limitations

There are several limitations of this study that should be considered. We used a database of admission-data level based on ICD-9-CM codification for which we might have overestimated bronchiolitis episodes by including early asthma and bronchitis or underestimated the frequency of bronchiolitis hospitalizations by not including misdiagnosed cases or those that developed bronchiolitis after hospitalization. This is an observational study based on aggregated and stratified data obtained for billing purposes. Changes in enrollment or discontinuation of private healthcare coverage by the insurer, as well as discrepancies in diagnostic-coding by physicians, hospitals, and post-adjustment measures for billing purposes could explain variations in bronchiolitis hospitalizations throughout the study period. The lack of individual patient characteristics and clinical information prevents us from determining and comparing annual incidence of bronchiolitis hospitalizations and its relationship with other risk factors and outcomes of interest, such as sociodemographic status, age-specific diagnosis, repeat hospitalization and PICU hospitalization, temporal distribution of bronchiolitis based on ICD-9-CM code, and presence of co-morbidities. Furthermore, we lacked information about the ethnicity of children included in the study but note that the study is based in a tropical country with a primarily Hispanic population. However, a small portion of children of non-Hispanic ethnicity might have been included in the study. The codification of the etiology within this study did not specify the method by which RSV laboratory testing was confirmed. Because there are differences in sensitivity and specificity for RSV testing, we may have over- or under-reported RSV hospitalizations. However, increases seen in the proportion of RSV hospitalizations were consistent with RSV outbreaks reported by the Puerto Rico Department of Health. We also lack information about influenza outbreaks, which could have affected the proportion of non-RSV bronchiolitis cases that were hospitalized. Because the insurer’s data on emergency visits and hospitalizations were mutually exclusive and analyzed separately, we cannot determine hospitalization rate after an emergency visit for bronchiolitis. Lastly, the study only included data from the private insurer. Procuring and including a representative sample that includes children covered by public health insurance or other insurance companies could strengthen future studies.

### Strengths

This is the first study to evaluate the epidemiological features of bronchiolitis in Puerto Rico by using representative data of a privately insured population between 2010 and 2014 from the largest insurer in Puerto Rico. This allowed us to determine population-based emergency department visits and hospitalization distributions due to bronchiolitis with decreased health disparities in comparison to public health insurance holders. November and December 2014 were the only months to overlap with the publication of the updated bronchiolitis management guidelines by the American Academy of Pediatrics, which allowed us to evaluate epidemiologic distributions without influence to providers’ decisions in hospitalizing children with bronchiolitis.

## Conclusions

Between 2010 and 2014, we found that the proportion of emergency department visits due to bronchiolitis is similar to that reported in the USA, while bronchiolitis hospitalizations among children in Puerto Rico was found to be twice as high. Further studies are needed to elucidate whether Hispanic ethnicity is a risk factor for increased frequency of hospitalization or is related to health disparities in the US healthcare system. Temporal trend shows year-round hospitalizations with an earlier seasonal peak and longer duration, consistent with Puerto Rico’s seasonal rainfall. This locally acquired data establishes the seasonality of bronchiolitis in Puerto Rico according to our epidemiology and helps us better define the pediatric population that has greater susceptibility so that interventions and prevention tools may be developed to prevent and control transmission of this disease in the tropical environment of Puerto Rico. Further studies are needed to elucidate whether this epidemiologic pattern can also be seen in publicly insured children and whether Hispanic ethnicity is a risk factor for increased hospitalizations or is related to health disparities in the US healthcare system.

## Abbreviations

RSV: Respiratory syncytial virus
